# Acrylamide formation in air-fried versus deep and oven-fried potatoes

**DOI:** 10.3389/fnut.2023.1297069

**Published:** 2024-01-11

**Authors:** Semra Navruz-Varlı, Hande Mortaş

**Affiliations:** Department of Nutrition and Dietetics, Gazi University, Ankara, Türkiye

**Keywords:** acrylamide, air fryer, deep frying, oven frying, food safety, home-fried potato

## Abstract

**Introduction:**

Present study investigates the effects of different home pre-treatment processes and cooking techniques on the acrylamide content of fried potatoes.

**Methods:**

Potato sticks were prepared in two different pre-treatment ways (washing and soaking) and cooked with three other techniques (air frying, deep frying, and oven frying). Acrylamide analyses were performed on cooked potatoes using an LC-MS/MS method.

**Results:**

The highest acrylamide content was found in potatoes cooked using the air fryer (12.19 ± 7.03 μg/kg). This was followed by deep frying (8.94 ± 9.21 μg/kg) and oven frying (7.43 ± 3.75 μg/kg). However, the difference between the acrylamide contents of the potatoes according to the cooking methods was not statistically significant. The acrylamide content of the potatoes that were subjected to soaking in all three ways was lower than the potatoes that were not soaked and only washed. In the deep-frying method, it was found statistically significant that the soaked potatoes contained less acrylamide (*p* = 0.029).

**Discussion:**

It is important to highlight the relatively low acrylamide levels found in oven-frying, lower than air frying in both washing and soaking groups in the present study. Although air fryers, which have become widely used as an alternative to deep frying in recent years, provide French fries with less oil, their role in the formation of acrylamide should be further investigated.

## 1 Introduction

Today, the increase in obesity and accompanying diseases such as diabetes, hypertension, and coronary heart diseases cause changes in cooking techniques and the creation of alternative food ingredients (1, 2). French fries, which are widely consumed, are also one of the dishes that get their share of alternative cooking techniques (3). It aims to reduce oil consumption and fat content of the potatoes by cooking French fries, which are classically prepared by frying in hot deep oil under normal atmospheric pressure, by cooking with alternative methods such as cooking in the microwave, oven, and air fryer (3, 4). However, chemical reactions in cooking processes cause the formation of some components, which are expressed as thermal process contaminants and adversely affect health (5). Acrylamide (2-propenamide), one of these components, is formed by the Maillard reaction because of heat treatment applications, especially in foods such as potato chips, French fries, and coffee (6, 7). The mechanism was explained to explain how acrylamide forms when the amino acid asparagine reacts with a carbonyl-containing compound at the usual cooking temperatures. This mechanism encompasses the creation of a Schiff base, followed by decarboxylation, and the elimination of either ammonia or a substituted imine due to heat, ultimately resulting in the production of acrylamide (8). It is vital to prevent its formation, as acrylamide is classified in Group 2A as probably carcinogenic to humans by the International Agency for Research on Cancer (IARC) (9).

Research continues on different applications to reduce acrylamide formation in fried potato strips (3, 4, 10, 11). The positive effects of other techniques on reducing acrylamide content are shown, including the use of various additives such as sodium acid pyrophosphate during processing (12), blanching, pre-treatments of the potatoes before cooking (13), and cooking using an air fryer instead of the traditional cooking method (3). Among these methods, the air fryer has started to be widely used today due to its practicality and rapid cooking (14).

In the air fryer method, potato slices are heated with oil droplets under hot air conditions, resulting in the drying and cooking of the potatoes (15). In this way, potatoes are fried with less oil. By providing a highly consumable final product in terms of taste, odor, color, and texture, 80% less oil absorption is achieved (16). The changes in acrylamide level caused by this cooking technique, which provides an advantage in reducing the amount of oil absorbed in fried potatoes, has been the subject of research recently (3, 17, 18). Giovanelli et al. (18) found that using air frying methods resulted in potatoes with high acrylamide (182 ± 24 μg/kg, using an acti-fryer) and low acrylamide (91 ± 13 μg/kg, with an air fryer) compared to deep frying (126 ± 15 μg/kg).

Similarly, in the study conducted by Verma et al. (3), it was shown that potatoes cooked with the air fryer technique had lower acrylamide content than microwave and deep soybean fat frying (270 μg/kg). However, the method differences found in these studies, including the use of frozen potatoes and uncertainty of the amount of oil used in the air fryer and acti-fryer (18), the addition of different extracts such as ginger (17), and the absence of soaking pre-treatment (3), are thought to affect the acrylamide level of the resulting product significantly. The limit of 500 μg/kg determined for French fries in the regulation numbered 22017/2158 published by the European Commission in 2017 can be used to implement procedures based on hazard analysis and critical control point (HACCP) principles (19). However, it is crucial to determine the maximum values for residues of potentially toxic compounds for institutional food services and to reveal the results obtained because of the pre-treatment and cooking techniques used at home (20). Therefore, there is a need to investigate the effects of home cooking practices on the acrylamide level in fried potato strips. This study aimed to determine whether the air frying method is an alternative to deep-frying and oven-frying in terms of acrylamide formation when cooking homemade potatoes with different pre-treatment processes.

## 2 Materials and methods

In this study, potato sticks were prepared with two different pre-treatment methods and cooked with three other frying methods. Acrylamide analyses were performed on cooked potatoes.

### 2.1 Pre-treatment of potatoes before cooking

Potatoes (*Solanum tuberosum*) were purchased from the Agricultural and Credit Cooperative Market Chain in Ankara, Türkiye, and stored for three days at 8–10°C until processing. After the potatoes were peeled using a mechanical potato peeling knife, 8 mm × 8 mm × 60 mm potato slices were obtained using an automatic potato slicer. Sliced potatoes were divided into two groups. The first group, which was determined as “soaking,” was kept in room temperature water (21°C) for 10 min, and the second group, which was determined as “washing,” was subjected to direct cooking after washing under running water for 30 s without applying the soaking process. With these processes, pre-treatment was prepared for potato strips designed at home with the classical method.

### 2.2 Frying methods

Different equipment was used for each of the three other cooking methods used in the study. A total of two different time-temperature combinations were applied, specific to the working principle of each equipment. The time-temperature combinations involved in the study were standardized before the research to create minimum time and temperature values at which the potatoes would turn golden yellow. In this study, since the air fryer, one of the new cooking methods that formed the basis for the planning of the research and aroused researchers’ curiosity in terms of acrylamide formation, was determined as the basic cooking method, the temperature-time combination (200°C-15 min) recommended by the manufacturer of the air fryer used in the study for French fries at half occupancy was preferred. Cooking degrees were determined after preliminary trials at the time and temperature selected for the air fryer, deep-frying, and oven-frying temperatures, and times that provide the closest cooking. In deep-frying, differently, the maximum frying oil temperature value (180 degrees) specified in the legislation is used to determine the temperature. In addition, since it is recommended by the air fryer manufacturer in the cooking procedure prepared for French fries to be cooked in the half-full chamber, the shake step was made at the 8th minute in the air frying. In other cooking techniques, stirring was done at the 8th minute to be simultaneous with the air frying technique. After stirring, the cooking process continued until the cooking time expired. The cooking time was determined as 15 min, 10 min, and 15 min in total for air frying, deep frying and oven frying, respectively, and the details of the cooking procedures were described in detail under the subheadings below.

**FIGURE 1:**
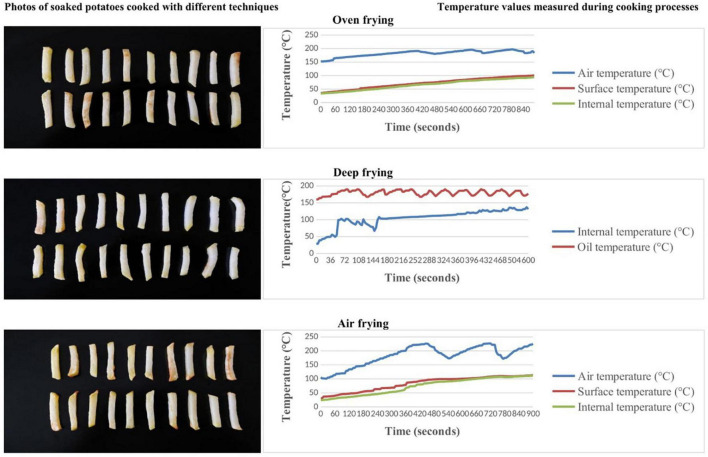
Time-temperature tracking and photos of potatoes with soaking pre-treatment process according to the different cooking techniques.

**FIGURE 2:**
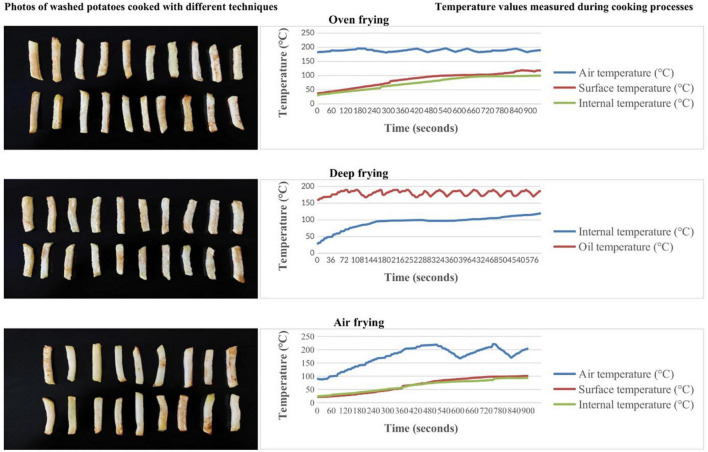
Time-temperature tracking and photos of potatoes with washing pre-treatment process according to the different cooking techniques.

Air, surface, and internal temperatures for air frying and oven; oil and internal temperatures for deep frying of potato strips were monitored using Extech Instruments SD200 3-Channel Temperature Datalogger. Medisana 48430 kitchen scale was used to weigh the samples. Cooking equipment, time-temperature combinations, and details of cooking processes are explained below. The temperatures at which the potatoes were kept during the cooking process and the photos of the cooked potatoes are shown in Figures 1, 2.

#### 2.2.1 Deep frying

Deep frying was carried out using a stainless-steel frying pan without a lid. Potato slices were fried at 180°C for 10 min. As deep-frying oil, 500 mL of sunflower oil was used for each experiment to submerge the potatoes in the oil completely. The potatoes were stirred once at the 8th minute during the cooking process. The thermometer verified this process by heating the oil to 180°C. The frying process was carried out in duplicate for the potatoes soaked in water at room temperature and for the potatoes that were only pre-washed, using new sunflower oil in each batch. The oil droplets remaining on the surface of the potatoes after deep frying were quickly removed with tissue paper. The deep-fried potato slices were put into 250 g sample packages and stored at −80°C until the analysis day, within a maximum of 2 h after they came to room temperature.

#### 2.2.2 Oven frying

Frying was performed in the oven (Siemens, Model Hb557jys0t, capacity 66 liters) at 200°C for 15 min in fan function. The oven was preheated at 200°C for 5 min in static function. Sunflower oil was used as two teaspoons (10 grams) per 250 grams of potato strips. The potato strips were placed in the middle of the oven after being arranged in a single row on a steel tray covered with greaseproof paper so that the potato slices were not touching each other. During the cooking process, at the 8th minute, the potatoes were taken out of the oven and flipped. The baking process was carried out in duplicate for the potato strips soaked in water at room temperature and for the potato strips that were only pre-washed. At the end of the time, the oven’s power was turned off, and potatoes were removed. Oil droplets are quickly removed with tissue paper. The baked potato strips were put into 250 g sample packages and stored at −80°C until the analysis day, within a maximum of 2 h after they came to room temperature.

#### 2.2.3 Air frying

A commercial air fryer (Mi Smart Air Fryer 3.5L, MAF02) was used in this research. The air fryer preheated for 5 min (reaching the temperature of 200°C) before inserting the potato strips. The potato strips were fried with 2 teaspoon sunflower oil (10 g) for every 250 g potatoes for 15 min at 200°C. During the cooking process, the potato chamber was removed at the 8th minute and the shaking process was performed. At the end of the time, the air fryer was turned off and the oil drops on the potatoes were quickly removed with a paper towel, and then kept on greaseproof paper to cool down to room temperature. The air-fried potato strips were put into 250 g sample packages and stored at −80°C until the analysis day.

### 2.3 Acrylamide analysis

Acrylamide analysis was carried out based on the method previously developed by Rufián-Henares and Morales (21). Agilent 6470A triple quadrupole LC-MS/MS system with Agilent MassHunter workstation software was used to determine the acrylamide concentration in French fries. After 1 g weight was taken from the homogenized sample, 10 mL of water was added and vortexed. After adding 10 mL of acetonitrile (ACN), it was shaken for another 10 min. After adding 1.3 to 1.5 g 4:1 (w:w) MgSO_4_:NaCl salt (Agilent QuEChERS pouch, p/n 5982-5550), it was vortexed again. It was centrifuged at 4,000 rpm for six min. 1 mL of the upper organic layer was drawn to a 2.0 mL microcentrifuge tube. 2 mL of the upper layer was drawn into a 15 mL centrifuge tube; the sample was evaporated in a water bath to dryness. 1 mL of water was added to the tube and vortexed. The sample was filtered through a 0.2 μm Agilent Captiva premium syringe filter (p/n 5190-5132). The supernatant was prepared for LC/MS injection using the positive ESI mode. The analysis procedure was carried out in accordance with the document Agilent Application Note for food testing and agriculture. The calibration curve was linear (*R*^2^ = 0.9986) with an equation of *y* = 0.004206 * x + 1.571242E-004. The diagnostic (LOD) and detection (LOQ) limits of the method were found to be 4.84 ng/g and 18.20 ng/g, respectively.

### 2.4 Statistical analysis

All statistical data were analyzed using IBM SPSS 22.0 (The Statistical Package for Social Sciences-SPSS Inc., Chicago, IL, USA). All data shown are the mean values of duplicates (*n* = 2) from two distinct runs. Descriptive analyses were presented using medians and interquartile range (IQR) for the non-normally distributed variables. As acrylamide contents of the potatoes were not normally distributed the Kruskal–Wallis tests were conducted to compare these parameters among cooking methods. The Mann–Whitney U test was performed to test the significance of pairwise differences using Bonferroni correction to adjust for multiple comparisons. The two values found in oven cooking (in soaking) and air frying (in soaking) were determined as outliers and were not included in the average calculation. The statistical significance was fixed at *p* ≤ 0.01 and *p* ≤ 0.05.

## 3 Results

The amount of acrylamide contained in potatoes according to the pre-treatment processes and cooking techniques is shown in Table 1. It was found that the acrylamide content of the potatoes that were subjected to soaking in all three methods, air frying (10.49 ± 8.86 μg/kg), deep frying (1.18 ± 0.18 μg/kg), and oven frying (5.87 ± 4.85 μg/kg), were lower than the potatoes that were not soaked and only washed (13.45 ± 6.45, 16.69 ± 6.12 and 8.59 ± 2.87 μg/kg, respectively). In the deep-frying method, it was found statistically significant that the soaked potatoes contained less acrylamide according to washing (*p* = 0.029).

**TABLE 1:** Acrylamide content of potatoes according to cooking techniques and pre-treatment procedure.

Cooking method	Pre-treatment	Acrylamide content (μ g/kg)			
		**Mean ± SD**	**M (IQR)**	*P*-values**	*P*-values***
Air frying	Soaking^a^	10.49 ± 8.86	9.57 (6.01)	*p* = 0.629	a-b, *p* = 0.057
	Washing^d^	13.45 ± 6.45	13.19 (12.27)		a-c, *p* = 0.700
Deep frying	Soaking^b^	1.18 ± 0.18	1.18 (0.35)	*p* = 0.029*	b-c, *p* = 0.057
	Washing^e^	16.69 ± 6.12	17.11 (11.71)		d-e, *p* = 0.686
Oven frying	Soaking^c^	5.87 ± 4.85	4.46 (3.15)	*p* = 0.400	d-f, *p* = 0.200
	Washing^f^	8.59 ± 2.87	8.95 (5.30)		e-f, *p* = 0.057

IQR, inter quantile range; SD, standard deviation. The superscript letters “a, b, c, d, e, f” indicate whether there is statistical significance for similar pre-treatments in different cooking methods.

*p < 0.05.

**The *P*-values expresses the difference between soaking and washing in the same cooking method.

***The *P*-values expresses the difference among different cooking methods in the same pretreatment category.

**FIGURE 3:**
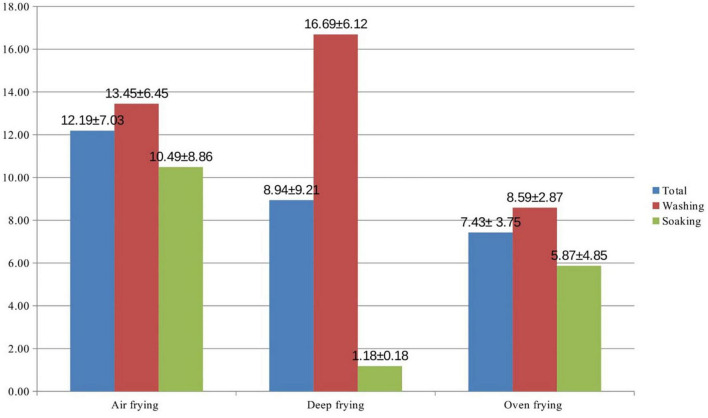
Acrylamide content of potatoes according to cooking techniques (mean ± standard deviation; *p* = 0.789).

A comparison of the acrylamide content of potatoes according to the different cooking methods is shown in Figure 3. Although the difference between the acrylamide contents of the potatoes according to the cooking methods was not statistically significant (*p* = 0.789), the highest acrylamide content was found in potatoes cooked using the air fryer (12.19 ± 7.03 μg/kg). This was followed by deep frying (8.94 ± 9.21 μg/kg) and oven frying (7.43 ± 3.75 μg/kg).

## 4 Discussion

This study aimed to investigate the acrylamide content of potatoes with different cooking and different pre-treatment techniques; it was shown that the potatoes with the highest acrylamide content were in potatoes cooked using the air fryer. However, it was shown that acrylamide content in potatoes cooked after 10 min of soaking in room temperature water decreased in all three cooking techniques, including air frying, deep frying, and oven frying.

Potatoes are widely consumed in Türkiye, like in other countries worldwide (22, 23), mostly as oil-fried (24), and in home-cooked conditions. Also, the rate of using air fryers for home-fried potatoes has increased noticeably in recent years in Türkiye as in many other countries. The most significant share in this increase can be stated as the practical use of hot air fryers and the cooking of potatoes using less oil. Since many companies around the world produce it, it has taken its place in the kitchens of thousands of houses with the effect of being at relatively affordable prices (15, 25, 26). However, there is insufficient awareness that this new generation of frying equipment may play a role in forming harmful compounds such as acrylamide (27). Acrylamide reduction strategies have successfully reduced exposure to industrially produced foods such as potato chips. Although home cooking processes have a high contribution to acrylamide exposure, little attention has been paid compared to industrial processes (28). Although studies are reporting that cooking potatoes with new cooking equipment, such as an air fryer, may be a new opportunity to reduce acrylamide exposure (3, 17, 28, 29), the European Food Safety Authority (EFSA) reports that hot air fryers produce 30–40% more acrylamide than conventional deep fryers (30). The lower acrylamide formation in the air fryer compared to deep frying has been attributed to differences in the amount of frying oil and oxygen during frying. Additionally, potatoes absorb up to 15% of oil during deep frying, which has been found to be associated with greater acrylamide formation in deep frying according to air fryer (17).

It is crucial to evaluate the benefits and risks of air fryers from the “risk-benefit approach” window. The need for more work in this area is highlighted (31). So, conducting this study is essential in terms of raising the Turkish population’s knowledge and awareness on the subject. In the present study, acrylamide levels were close to the values in the survey by Palazoğlu et al. while lower than in other studies (3, 17, 29, 32–34). Possible factors that may cause this result are (i) differences in the types of potatoes used (lower acrylamide formation is expected in potatoes with low reducing sugar content), (ii) differences in frying time and temperatures (due to the nature of the frying process, an increase in frying temperature results in a reduction in frying time and a decrease in frying temperature results in an increase in frying time. Different temperature-time combinations in different studies affect the amount of acrylamide formation.), (iii) differences in slice thickness (thin, very thin or thick slices), (iv) differences in pretreatment methods, and (v) differences in the type of frying oil used.

Palazoğlu et al. (32) noted that in baking, the acrylamide level was the highest in potato chips baked at 170°C compared to 180°C and 190°C. This interesting result showed that low-temperature baking may indeed result in higher acrylamide levels. Air fryers can also be used as an alternative to ovens due to their more practical use. For air fryers produced by different companies, the time-temperature recommendations for French fries differ (some suggest lower temperatures, while others may recommend higher temperatures, such as 200 degrees). Verma et al. (3) reported that acrylamide concentration differed significantly between frying types, interestingly, regardless of time-temperature combinations. While acrylamide formation increases with time and temperature in deep frying, it is minimized by combining high temperature and short time (200°C, 8 min) in an air-fryer (3). Air fryer manufacturers should objectively determine the most appropriate cooking temperatures and times for foods with a high risk of acrylamide formation, especially for potatoes, which are widely consumed by all age groups starting from the early childhood age group. While the producer companies are doing this, the type of potato, the use of fresh or frozen potatoes, different cutting shapes, different thinness-thicknesses, the kind of oil to be used, etc., must consider all factors. Most importantly, companies should inform their customers about the risk of acrylamide formation in case of improper use, using an appropriate communication language that is simple enough for consumers of all education levels to understand.

Deep frying has been reported to cause higher levels of acrylamide formation in potatoes compared to baking (32, 33). In the present study, the highest and lowest concentrations of mean acrylamide were found in the air-fryer and oven-frying methods, respectively. The similar difference is between washing and soaking (higher and lower, respectively) in all methods (*p* > 0.05). This is a similar finding to studies reporting that frying results in higher acrylamide concentrations than baking potatoes (17, 34). It is important to highlight also the relatively low acrylamide levels found in oven-frying, lower than air frying in both washing and soaking groups in the present study. It is also a contrasting finding to studies reporting higher acrylamide formation with deep frying than air fryers (35, 36). In the present study, the higher levels of acrylamide in air-fried potatoes compared to deep-fried potatoes are thought to be since the frying temperature. While the frying oil temperature reached a maximum of 190 degrees in deep frying, there were moments when the air temperature reached 229 degrees in the air fryer (shown in Figures 1, 2).

The average oil temperature and standard deviation during the 10 min the potatoes were fried was determined as 180.07 ± 7.26 degrees. After the frying oil was heated to 180 degrees and the potatoes were added to the oil, the oil temperature decreased slightly and then increased again. It is thought that the fluctuations in oil temperature during frying may be due to the water vapor released from the potatoes reducing the oil temperature. Because it was observed that the potato internal temperature was lower than the frying oil temperature throughout the frying process (average potato internal temperature value is 93.32 ± 19.56 degrees). As literature information supporting this statement, in determining the thermal input to which potato slices are exposed, it is recommended to use the potato slice temperature rather than the frying oil temperature (32).

It should be noted that the air-fryer may not be a suitable substitute for some deep-fried foods, as some foods may have a different texture or flavor than traditional deep-fried (36). In the present study, almost none of the potatoes cooked in the air-fryer had texture and the sensory characteristics of deep-fried potatoes (such as desired crispness, taste, and aroma), although an objective examination of sensory characteristics was not performed in the present study. To provide the selected sensory properties in the potatoes cooked in the air-fryer, the potatoes should be cooked longer times (15, 25) because the heat transfer rate is much slower in a gas phase (oil mist) than in liquid phase (bulk oil) (15, 25), and this increases the risk of acrylamide formation. In a study, it was found that French fries obtained by air frying (using an acti-fry) contain higher acrylamide and poor quality characteristics according to the deep frying (18). In addition, when cooking potatoes in an air fryer under home conditions, consumer habits and preferences regarding texture and the organoleptic properties of the fried potato will also come into play. Therefore, there may be exposure to much more than the average amount of acrylamide detected in potatoes cooked in the air fryer in the present study.

In order to reduce the adverse effects of acrylamide on health, it is recommended to apply pre-treatments such as soaking potatoes before frying (19). In the present study, the acrylamide content of the potatoes that were subjected to soaking in all three methods was lower than the potatoes that were not soaked and only washed (only the difference in the deep frying method was statistically significant). Acrylamide formation is reduced when fresh potatoes cut at home are kept in room temperature water for 10 min as a straightforward application, so it is thought that exposure can be reduced by raising awareness of society on this issue.

In the present study, potatoes cooked after soaking were found to contain lower acrylamide in each cooking technique compared to potatoes cooked after washing only. Additionally, it has been shown that the relatively low acrylamide levels found in oven-frying, lower than air frying in both washing and soaking groups in the present study. Essential findings were obtained that can be used to develop recommendations for consumers to minimize acrylamide exposure, which is one of the chemical hazards arising from heat treatment in potatoes, and to ensure the production of safer and higher quality fried potatoes. These findings will raise awareness about choosing cooking methods and equipment in the home setting when choosing a frying method for French fries, which will limit the health benefits associated with offering less oil and keep chemical hazards to a minimum. However, more research is still needed to confirm these findings and explore the potential health effects of each frying method—particularly for next-generation frying equipment. Training on understanding acrylamide exposure from home cooking practices and strategies to reduce exposures should be increased.

## 5 Strengths and limitations of the study

The most potent aspect of the study is that it gives society a perspective on the risks associated with acrylamide formation when frying potatoes in air fryers. When potatoes were soaked in water at room temperature for 10 min before deep-frying, acrylamide formation was significantly reduced compared to potatoes that were not soaked in water. Based on this finding, strength of the study is that a practical suggestion has been developed to reduce acrylamide formation in deep-frying, one of society’s most frequently preferred frying methods.

The limitations of this study are that only the acrylamide content in homemade fried potatoes was examined and that various indicators that are important in comparing processing contaminants such as reducing and total sugars, asparagine and oil content of potatoes and sensory quality parameters were not examined. The effect of cooking methods on physicochemical properties and quality attributes such as moisture, texture, color, flavor, oil and sugar content should also be evaluated in future studies. Secondly, in the study, each processing procedure was applied in four different groups, totally 24 samples, acrylamide analyzes were performed on cooked potato samples, and mean and standard deviation values were presented. For this reason, it is thought that the standard deviation values given in the study are high and the standard deviation values will be lower if the study is conducted with more samples in the further studies.

## Data availability statement

The raw data supporting the conclusions of this article will be made available by the authors, without undue reservation.

## Author contributions

SN-V: Conceptualization, Funding acquisition, Writing – original draft, Writing – review & editing, Methodology, Supervision. HM: Conceptualization, Data curation, Funding acquisition, Methodology, Writing – original draft, Writing – review & editing, Formal analysis.
